# Gordon Hospital

**Published:** 1902-04-26

**Authors:** 


### GORDON HOSPITAL.

### A SELF-SUPPORTING INSTITUTION, SAVE FOR ITS DEBT.

The eighteenth annual meeting of the Gordon Hospital for Fistula, Vauxhall Bridge Road, on the 10th instant, was of the customary quiet and uneventful character, the 12 governors present being all members of the general committee or of the surgical staff. Captain A. S. Hincks presided and moved the adoption of the report, which was agreed to, as were votes of thanks to the staff and honorary officers, and also a resolution appointing Messrs. Sutton Ommanney and Rendall honorary solicitors in the room of Mr. Murray, whose resignation through ill-health the committee had received with regret. Mr. Murray was also chairman of the committee, of which he had been a member from the foundation of the institution. This hospital is a special one, not only with regard to the class of disease dealt with, but also owing to the fact that its main object, as set forth in the report, is to provide for the needs of those who can hardly with propriety avail themselves of a free hospital, yet find the cost of private treatment too great a strain upon their resources. The hospital contains 10 wards. Of these, three wards are single-bedded, the charge for which is three guineas per week ; four wards contain two beds each, the charge for which is two guineas per week ; the remaining wards contain from three to six beds each, and are intended for the accommodation of those whose means do not permit of their paying. The members of the surgical staff give their services free to the institution, and the whole of the payments made by the patients is devoted to their maintenance while they are in the hospital, and to the support of the hospital itself.

The number of patients seeking the benefits of the hospital last year showed an increase over previous years—295 patients having been admitted, as against 289 in the year 1900. That the single-bedded wards are appreciated is proved by the fact that 52 patients availed themselves of them, whereas only 37 patients were admitted into them in the year 1900.

The receipts for the year were £1,323 paid by patients, and £246 from subscriptions and donations. Both these sums showed a falling off as compared with last year.

The committee again draw attention to the heavy building debt of £6,000 with which the hospital is encumbered, and express the hope that friends will assist them in wiping out this debt, but for which the hospital would be nearly self-supporting.

